# Diagnostic Accuracy of ^123^I-Meta-Iodobenzylguanidine Myocardial Scintigraphy in Dementia with Lewy Bodies: A Multicenter Study

**DOI:** 10.1371/journal.pone.0120540

**Published:** 2015-03-20

**Authors:** Mitsuhiro Yoshita, Heii Arai, Hiroyuki Arai, Tetsuaki Arai, Takashi Asada, Hiroshige Fujishiro, Haruo Hanyu, Osamu Iizuka, Eizo Iseki, Kenichi Kashihara, Kenji Kosaka, Hirotaka Maruno, Katsuyoshi Mizukami, Yoshikuni Mizuno, Etsuro Mori, Kenichi Nakajima, Hiroyuki Nakamura, Seigo Nakano, Kenji Nakashima, Yoshiyuki Nishio, Satoshi Orimo, Miharu Samuraki, Akira Takahashi, Junichi Taki, Takahiko Tokuda, Katsuya Urakami, Kumiko Utsumi, Kenji Wada, Yukihiko Washimi, Junichi Yamasaki, Shouhei Yamashina, Masahito Yamada

**Affiliations:** 1 Department of Neurology and Neurobiology of Aging, Kanazawa University Graduate School of Medical Science, Kanazawa, Ishikawa 920–8640, Japan; 2 Department of Neurology, Hokuriku National Hospital, Nanto, Toyama 939–1893, Japan; 3 Department of Psychiatry, Juntendo University School of Medicine, Bunkyo, Tokyo 113–8431, Japan; 4 Institute of Development, Aging and Cancer, Tohoku University, Sendai, Miyagi 980–8575, Japan; 5 Department of Neuropsychiatry, Institute of Clinical Medicine, University of Tsukuba, Tsukuba, Ibaragi 305–8576, Japan; 6 PET/CT Dementia Research Center, Juntendo Tokyo Koto Geriatric Medical Center, Juntendo University School of Medicine, Koto, Tokyo 136–0075, Japan; 7 Department of Geriatric Medicine, Tokyo Medical University, Shinjuku, Tokyo 160–0023, Japan; 8 Department of Behavioral Neurology and Cognitive Neuroscience, Tohoku University Graduate School of Medicine, Sendai, Miyagi 980–8575, Japan; 9 Department of Neurology, Okayama Kyokuto Hospital, Okayama, Okayama 703–8265, Japan; 10 Medical Care Court Clinic, Yokohama, Kanagawa 225–0014, Japan; 11 Department of Radiology, Toranomon Hospital, Minato, Tokyo 105–8470, Japan; 12 Graduate School of Comprehensive Human Sciences, University of Tsukuba, Bunkyo, Tokyo 112–0012, Japan; 13 Department of Neurology, Division of Neurogenerative Medicine, Kitasato University School of Medicine, Sagamihara, Kanagawa 252–0374, Japan; 14 Department of Nuclear Medicine, Kanazawa University Hospital, Kanazawa, Ishikawa 920–8640, Japan; 15 Department of Environmental and Preventive Medicine, Graduate School of Medical Science, Kanazawa University, Kanazawa, Ishikawa 920–8640, Japan; 16 Center for Treatment, Care and Research of Dementia, Medical Co. LTA, Sumida, Tokyo 130–0004, Japan; 17 Division of Neurology, Department of Brain and Neurosciences, Faculty of Medicine, Tottori University, Yonago, Tottori 683–8504, Japan; 18 Department of Neurology Kanto Central Hospital of the Mutual Aid Association of Public School Teachers, Setagaya, Tokyo 158–8531, Japan; 19 Tokai Central Hospital, Kakamigahara, Gifu 504–0816, Japan; 20 Department of Molecular Pathobiology of Brain Diseases, Graduate School of Medical Science, Kyoto Prefectural University of Medicine, Kyoto, Kyoto 602–0841, Japan; 21 Department of Biological Regulation, School of Health Science, Faculty of Medicine, Tottori University, Yonago, Tottori 683–8503, Japan; 22 Department of Neuropsychiatry, Sunagawa City Medical Center, Sunagawa, Hokkaido 073–0196, Japan; 23 Department for Cognitive Disorders, Hospital of National Center for Geriatrics and Gerontology, Obu, Aichi 474–8511, Japan; 24 Division of Cardiovascular Medicine, Department of Internal Medicine, Ohmori Hospital, Toho University School of Medicine, Ota, Tokyo143–8541, Japan; Philadelphia VA Medical Center, UNITED STATES

## Abstract

**Background and Purpose:**

Dementia with Lewy bodies (DLB) needs to be distinguished from Alzheimer’s disease (AD) because of important differences in patient management and outcome. Severe cardiac sympathetic degeneration occurs in DLB, but not in AD, offering a potential system for a biological diagnostic marker. The primary aim of this study was to investigate the diagnostic accuracy, in the ante-mortem differentiation of probable DLB from probable AD, of cardiac imaging with the ligand ^123^I-meta-iodobenzylguanidine (MIBG) which binds to the noradrenaline reuptake site, in the first multicenter study.

**Methods:**

We performed a multicenter study in which we used ^123^I-MIBG scans to assess 133 patients with clinical diagnoses of probable (n = 61) or possible (n = 26) DLB or probable AD (n = 46) established by a consensus panel. Three readers, unaware of the clinical diagnosis, classified the images as either normal or abnormal by visual inspection. The heart-to-mediastinum ratios of ^123^I-MIBG uptake were also calculated using an automated region-of-interest based system.

**Results:**

Using the heart-to-mediastinum ratio calculated with the automated system, the sensitivity was 68.9% and the specificity was 89.1% to differentiate probable DLB from probable AD in both early and delayed images. By visual assessment, the sensitivity and specificity were 68.9% and 87.0%, respectively. In a subpopulation of patients with mild dementia (MMSE ≥ 22, n = 47), the sensitivity and specificity were 77.4% and 93.8%, respectively, with the delayed heart-to-mediastinum ratio.

**Conclusions:**

Our first multicenter study confirmed the high correlation between abnormal cardiac sympathetic activity evaluated with ^123^I-MIBG myocardial scintigraphy and a clinical diagnosis of probable DLB. The diagnostic accuracy is sufficiently high for this technique to be clinically useful in distinguishing DLB from AD, especially in patients with mild dementia.

## Introduction

Ante-mortem diagnosis of dementia with Lewy bodies (DLB) and differentiating it from Alzheimer’s disease (AD) are important to determine prognosis and better management [1, 2]. Some patients with DLB have an accelerated disease progression and respond well to cholinesterase inhibitors, and approximately half of the patients experience life threatening adverse reactions to antipsychotic medications [3, 4]. The number of cases is expected to increase as the population ages and as DLB becomes increasingly recognized in the differential diagnosis of dementia [5, 6]. Consensus clinical diagnostic criteria have high (80–90%) specificity but low sensitivity even in specialist research settings when compared with neuropathological autopsy findings [7, 8]. Institutions in non-specialist clinical settings are likely to be even more imperfect for the diagnosis of DLB. The most common misdiagnosis reported in these studies was AD [7–9].

Meta-iodobenzylguanidine (MIBG) is a physiologic analogue of noradrenaline, used to determine the location, integrity, and function of postganglionic noradrenergic neurons. ^123^I-MIBG cardiac scintigraphy is a noninvasive tool for estimating local myocardial sympathetic nerve damage in various heart and neurologic diseases [10–12]. Noradrenergic post-ganglionic sympathetic denervation is a common feature of Parkinson’s disease (PD) and related Lewy body disorders. Patients with PD can exhibit reduced cardiac ^123^I-MIBG accumulation without evidence of other autonomic failure, whereas those with DLB can have reduced cardiac ^123^I-MIBG uptake without evidence of parkinsonism [12, 13]. Recently, markedly reduced cardiac MIBG uptake in idiopathic rapid eye movement sleep behavior disorder consistent with the loss of sympathetic terminals was reported, and an association of Lewy body pathology was suggested [14].

As ^123^I-N-ω-fluoropropyl-2β-carbomethoxy-3β-(4-iodophenyl) nortropane (FP-CIT) single photon emission computed tomography (SPECT) successfully visualizes presynaptic dopaminergic degeneration of the nigrostriatal tract, the finding of reduced tracer uptake in the basal ganglia is recognized as a suggestive feature of DLB [15]. The study to differentiate PD from atypical parkinsonian disorder using both ^123^I-FP-CIT SPECT and MIBG scintigraphy revealed that diagnostic accuracy was similar in both methods [16, 17]. However, there have been no multicenter studies that established diagnostic accuracy of ^123^I-MIBG SPECT imaging.

The primary aim of this multicenter study was to determine the diagnostic accuracy of ^123^I-MIBG imaging in the ante-mortem differentiation of DLB from AD. Furthermore, we examined the diagnostic accuracy in mild dementia cases, because differential diagnosis of dementia in early stage is difficult and important [7–9]. Our study confirmed the high correlation between abnormal cardiac sympathetic activity evaluated with ^123^I- MIBG cardiac scintigraphy and a clinical diagnosis of probable DLB. The diagnostic accuracy was sufficiently high for this technique to be clinically useful in distinguishing DLB from AD, especially in patients with mild dementia, indicating a significant contribution of ^123^I-MIBG imaging to increasing the diagnostic accuracy of DLB.

## Methods

### Subjects

Between July 2010 and December 2011, we performed a multicenter study in 10 Japanese sites. We included patients aged 55–85 years who met at least one of the following: consensus criteria for probable or possible DLB [15], National Institute of Neurological and Communicative Disorders and Stroke-Alzheimer’s Disease and Related Disorders Association (NINCDS-ADRDA) criteria for probable AD [18]. A mini-mental state examination (MMSE) [19] score of 10 or more was required to ensure that patients could complete sufficient assessments to provide useful diagnostic information. We defined patients with dementia who had developed parkinsonism more than a year before onset of dementia symptoms as having PD with dementia [4] and excluded them from the study. We also excluded patients who met the exclusion criteria of ^123^I-MIBG scintigraphy as follows: (1) patients taking tricyclic antidepressants and/or reserpine, (2) patients with cardiac failure, (3) patients who had ischemic heart disease within six months of participation, (4) patients who had myocardial blood flow SPECT abnormalities within one year of participation, (5) patients planning to have surgeries of major arteries including revascularization within two months of participation, (6) patients with poorly controlled diabetes mellitus [HbA1c > 7.0%] or receiving insulin therapy, (7) patients with severe kidney dysfunction or renal failure [eGFR < 15 mL/min/1.73m^2^], (8) patients receiving hemodialysis, (9) patients with pheochromocytoma, (10) patients with amyloid neuropathy or other obvious peripheral neuropathy, (11) patients with a history of neoplasm within five years of participation, and (12) patients being pregnant, nursing, or having possibility of pregnancy. Other exclusion criteria were: (1) PD, cerebral infarction that affects cognitive function, Huntington disease, normal pressure hydrocephalus, brain tumor, progressive supranuclear palsy, epilepsy, subdural hematoma, multiple sclerosis, or head injury with aftereffect, (2) patients with infection or focal regions revealed by MRI such as cerebral infarction that affects cognitive function, (3) patients with a cardiac pacemaker, aneurysm clips, prosthetic valves, cochlear implants, or other metal implants, (4) patients with a history of alcohol or drug abuse, severe or unstable disease, deficiency of vitamin B12 or folic acid, syphilis, or thyroid dysfunction, and (5) patients judged as inappropriate by a clinical evaluation committee.

### Ethics

The study was done in accordance with the current revision of the Declaration of Helsinki and applicable to national and local laws and regulations. All patients and their caregivers gave written informed consent. This study was approved by the Medical Ethics Committee of Kanazawa University and also by institutional review boards of all participating centers (S1 Table).

### Study protocol

Clinical diagnosis was established by an independent consensus panel, consisting of three clinicians (experts in the field of DLB), who were provided with a patient profile stemming from quality-assured clinical data from the onsite investigators’ case record forms and copies of onsite original source data, containing full details of the following neuropsychiatric assessments: MMSE [19], the investigator’s estimation of the geriatric depression scale, neuropsychiatric inventory [20], clock drawing test, and clinical dementia rating [21]. Results of MRI scans and the onsite investigators’ clinical diagnosis before imaging were also available. The consensus panel did not have access to ^123^I-MIBG scintigraphy findings at any stage and was unaware of the patients’ identities and initials, and names of institutions and investigators.

This study is also registered as UMIN000003419 (http://www.umin.ac.jp/). Recruitment and enrollment began in 01.07.2010 and follow-up testing was completed 31.12.2011.

### ^123^I-MIBG myocardial scintigraphy

Within a month of clinical diagnosis, planar and SPECT images were acquired at 20–30 min and 3–4 hr after a single intravenous injection of 111MBq ^123^I-MIBG (supplied by Fujifilm RI Pharma, Co. Ltd, Tokyo, Japan). To obtain scintigraphic images the energy discrimination was centered on 159 keV with a 20% window. All the institutions used standard acquisition conditions, and normal values of the heart-to-mediastinum (H/M) ratio are described elsewhere [22, 23].

**Fig 1:**
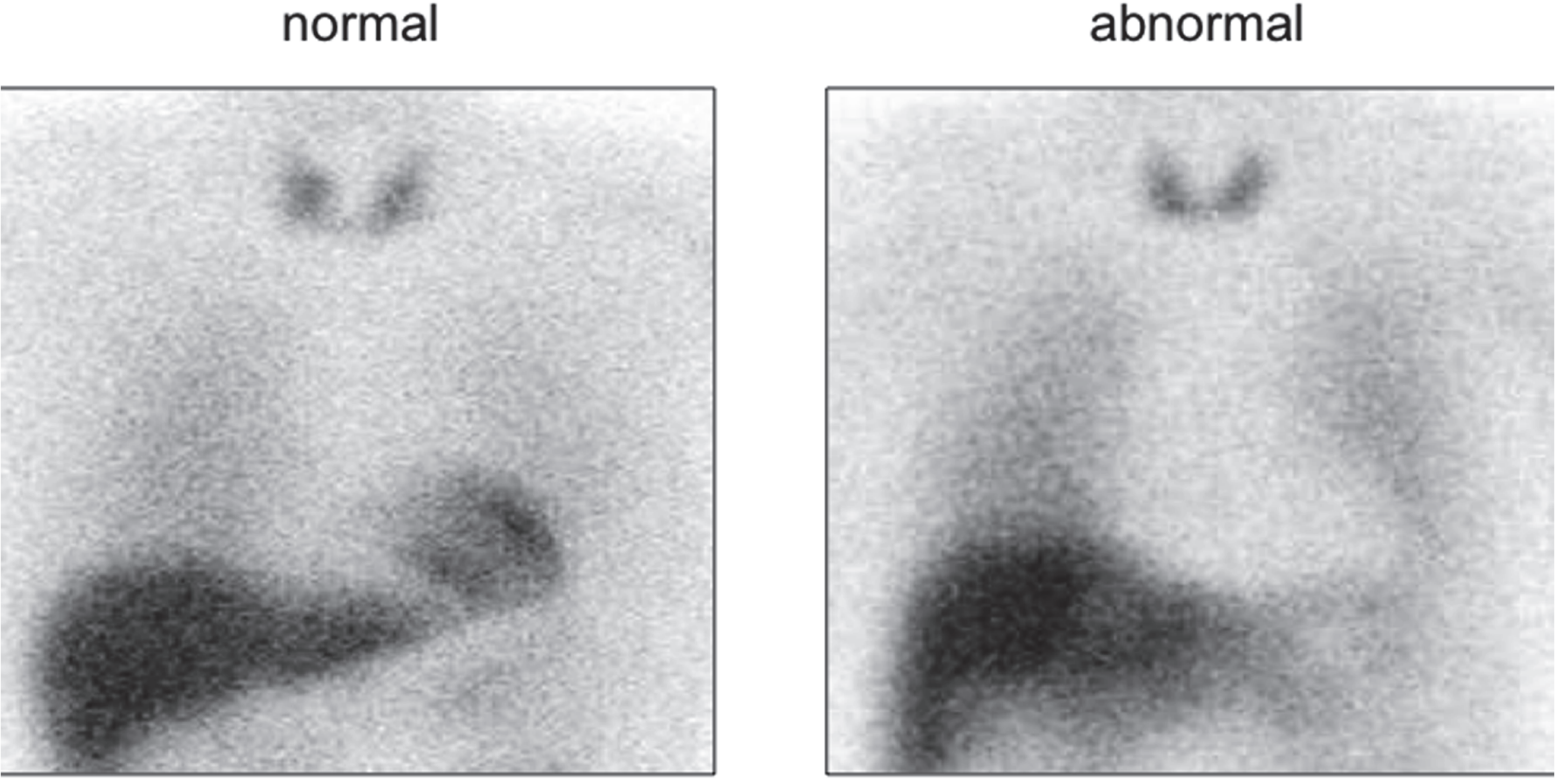
Normal and abnormal planar image of ^123^I-MIBG cardiac scintigraphy.

Anterior planar imaging was required for the quantification of the H/M ratio. All the MIBG images were sent to an independent image review center (Kanazawa, Japan). The H/M ratio was calculated using a standard method, by dividing the average count per pixel in the circular region of interest (ROI) on the heart by that in the rectangular ROI on the upper mediastinum. An MIBG software program that can provide automated ROI-based semi-quantification of H/M ratio was used in this study [24]. The algorithm of this software includes cross calibration of H/M ratios among hospitals that is caused by the differences in collimator types. The cross-calibration was based on the phantom studies in all hospitals, and a H/M ratio obtained from a low-energy type collimator was converted to a value comparable to a medium-energy type collimator [24]. Aside from the H/M ratio, three independent blinded physicians with expertise in MIBG imaging assessed myocardial MIBG uptake visually and classified the cardiac MIBG activity into four grades; namely, grade 0:normal, grade 1:probably normal, grade 2: probably abnormal and grade 3: abnormal. All three readers interpreted the planar images firstly and subsequently with addition of SPECT images in a random order. The readers finally classified the images as either normal (grades 0 and 1) or abnormal (grades 2 and 3) (Fig. 1).

### Statistical analysis

We analyzed the data with JMP 10.0.2 (SAS Institute Inc., Cary, NC, USA). For binomially distributed data, we assessed differences among the different diagnostic groups (probable DLB, possible DLB, probable AD) with respect to patients’ characteristics by means of χ^2^ tests. We used an analysis of variance (ANOVA) for normally distributed data; if normality could not be established, we used the non-parametric Kruskal-Wallis test. Our primary analysis was a comparison of the H/M ratio and results of visual assessment (normal or abnormal scan) in patients with probable DLB or probable AD. For this analysis, we calculated: sensitivity—the percentage of times that the image diagnosis was abnormal given that the clinical diagnosis was probable DLB; specificity—the percentage of times that the image diagnosis was normal given that the clinical diagnosis was probable AD; accuracy—the percentage of times the image diagnosis matched the clinical diagnosis; positive predictive value (PPV)—the percentage of times that the clinical diagnosis was probable DLB given that the image diagnosis was abnormal; and negative predictive value (NPV)—the percentage of times that the clinical diagnosis was probable AD given that the image diagnosis was normal. We calculated 95% CIs for these estimates with the Wilcocson score method. Sample size calculations were based on the hypothesis that the sensitivity and specificity rates of ^123^I-MIBG imaging in the detection of probable DLB and probable AD patients would be 99% and 98%, respectively (based on an earlier single site study) [13, 25]. Using a one-sided, one-sample χ^2^ test with a target significance level of 0.025, a total of 101 patients (55 DLB and 46 AD) were needed to achieve 90% power to detect -0.10 (sensitivity) and -0.13 (specificity) difference between these anticipated targets and prespecified thresholds (0.89 for sensitivity, 0.85 for specificity). An over-enrolment of 10% was done to adjust for incorrect clinical categorization requiring approximately 110 patients to be enrolled. Additional 30 possible DLB cases were enrolled to allow a secondary objective of assessing the performance of imaging in this group for three years follow up (a total of at least 140 subjects). We ascertained inter-reader agreement for visual assessment (normal or abnormal scan) with Cohen’s κ statistic for each pair of independent image readers. Additionally, we calculated a generalized κ coefficient that simultaneously combined the results of all three independent readers. κ values are equal to zero when the agreement does not differ from chance and equal to one when there is perfect agreement.

To gain further insight into the mild dementia cases, a cut-point of 21/22 of the MMSE score was applied to assign both probable DLB and probable AD patients to mild (n = 47) and moderate/severe (n = 60) dementia [19]. We evaluated the sensitivity and specificity to distinguish probable DLB from probable AD in both mild (MMSE ≥ 22) and moderate/severe (MMSE ≤ 21) dementia groups.

A receiver operating characteristic (ROC) curve for the prediction of DLB was created using H/M ratio as the predictor. The results are expressed as mean values ± SD. Values with *p* < 0.01 were regarded as significant.

## Results

**Fig 2:**
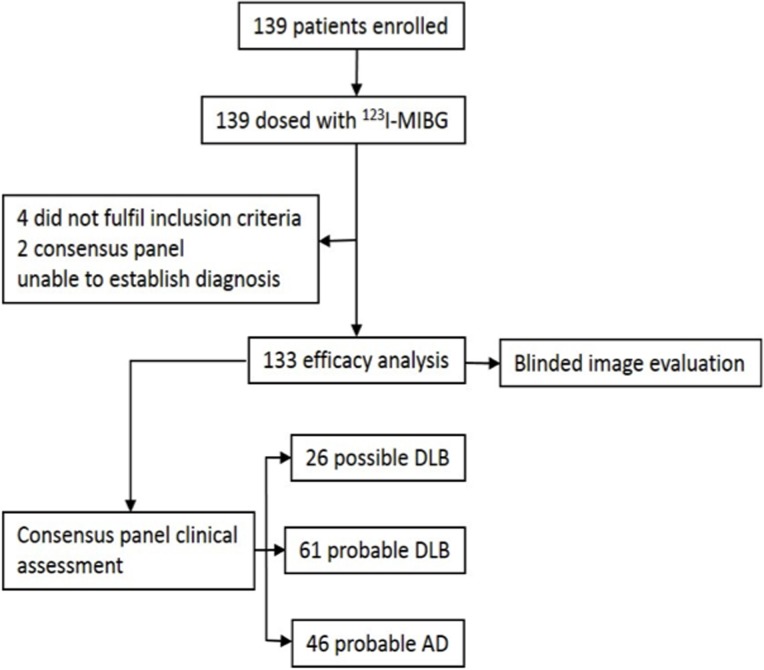
Flow diagram of the eligible patients and the enrolling process of the study.

Of the 139 individuals who were enrolled and received ^123^I-MIBG, four subjects who did not fulfill the inclusion criteria and two subjects whose clinical diagnosis was not established by the expert consensus panel were excluded (Fig. 2). The patients’ characteristics are shown in Table 1. The mean age of the 133 patients who were included in the efficacy analysis was 76.0 ± 6.2 years and 42.8% were men. Sixty one of the patients were diagnosed with probable DLB, 26 with possible DLB, and 46 with probable AD. All cases had MRI structural scans as part of the diagnostic procedure. Table 2 shows the results of the three blinded image readers with respect to the visual assessment findings—probable DLB versus probable AD patients. The mean sensitivity of ^123^I-MIBG with both planar and SPECT imaging for a clinical diagnosis of probable DLB was 68.9% (95% CI 56.4–79.1) and specificity 87.0% (74.3–93.9). We obtained values of 76.6% (67.8–83.6) for accuracy, 87.5% (75.3–94.1) for PPV, and 67.8% (55.1–78.3) for NPV. The inter-reader agreement regarding visual assessment (normal or abnormal scan) was very high between the independent readers A and B: 0.75 (95% CI 0.65–0.84); A and C: 0.84 (0.75–0.92); B and C: 0.88 (0.81–0.95). For all three readers simultaneously, Cohen’s κ was 0.82 (0.75–0.88), which also indicates good agreement.

**Table 1:** Clinical characteristics of the subjects.

	Probable DLB (n = 61)	Possible DLB (n = 26)	Probable AD (n = 46)	*p value*
Sex (M/F)	28/33	12/14	17/29	0.607*
Age (y)	76.9 (5.4)	76.4 (6.0)	74.5 (7.3)	0.151†
MMSE	20.5 (5.6)	20.2 (4.0)	19.7 (4.9)	0.719†
CDR-J	1.25 (0.76)	1.17 (0.66)	1.17 (0.63)	0.802†
CDT	2.6 (1.6)	3.5 (1.5)	3.0 (1.8)	0.071†
Hoehn & Yahr	1.64 (1.29)	0.54 (0.95)	0	< 0.0001‡

Data are mean (SD).

*χ^2^ test.

†ANOVA.

‡Kruskal-Wallis test.

MMSE: mini-mental state examination; CDR-J: clinical dementia rating scale-Japan; CDT: clock drawing test.

**Table 2:** Sensitivity and specificity of visual assessment in differentiating between probable DLB and probable AD.

	Sensitivity (95% CI)	Specificity (95% CI)	Accuracy (95% CI)	PPV (95% CI)	NPV (95% CI)
Reader A	65.6 (53.0–76.3)	87.0 (74.3–93.9)	74.8 (65.8–82.0)	87.0 (74.3–93.9)	65.6 (53.0–76.3)
Reader B	68.9 (56.4–79.1)	87.0 (74.3–93.9)	76.6 (67.8–83.6)	87.5 (75.3–94.1)	67.8 (55.1–78.3)
Reader C	68.9 (56.4–79.1)	87.0 (74.3–93.9)	76.6 (67.8–83.6)	87.5 (75.3–94.1)	67.8 (55.1–78.3)
Majority Vote	68.9 (56.4–79.1)	87.0 (74.3–93.9)	76.6 (67.8–83.6)	87.5 (75.3–94.1)	67.8 (55.1–78.3)

**Fig 3:**
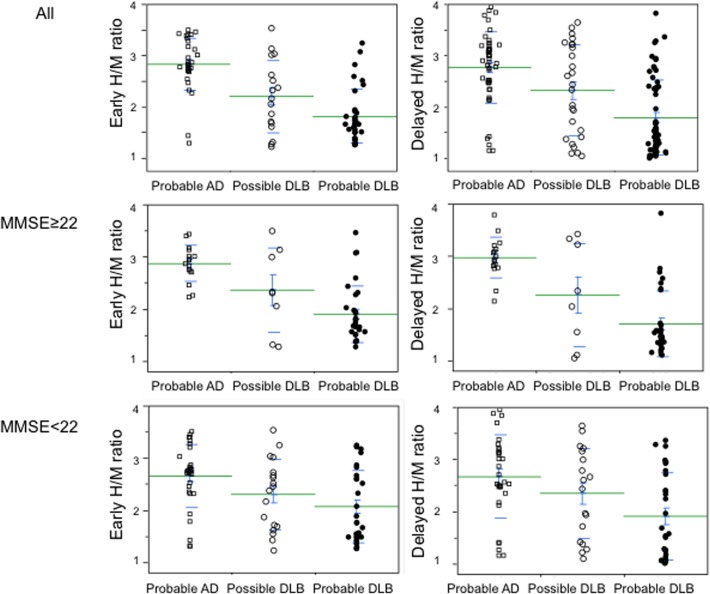
Individual values for the H/M ratio of ^123^I-MIBG uptake. Significant reductions in early and delayed H/M ratios were observed in probable DLB compared with probable AD group of all cases, mild dementia cases (MMSE ≥ 22), and moderate/severe dementia cases (MMSE ≤ 21) (see text). Green lines indicate the mean value of H/M ratio. AD: Alzheimer’s disease; DLB: dementia with Lewy bodies.

The H/M ratio was lower in the probable DLB group (early: 1.97 ± 0.62; delayed: 1.79 ± 0.73) than that in the possible DLB group (early: 2.32 ± 0.71, *p* = 0.0424; delayed: 2.32 ± 0.88, p = 0.0087) and the probable AD group (early: 2.72 ± 0.54, *p* < 0.0001; delayed: 2.77 ± 0.70, *p* < 0.0001) (Fig. 3). There were no significant difference between the groups of probable AD and the possible DLB (early: *p* = 0.0200; delayed: *p* = 0.00414). The group of patients with mild dementia consisted of 16 with probable AD, 8 with possible DLB, and 31 with probable DLB. In the mild dementia group, the H/M ratio was lower in the probable DLB group (early: 1.90 ± 0.54; delayed: 1.70 ± 0.63) than that in the probable AD group (early: 2.86 ± 0.35, *p* < 0.0001; delayed: 2.97 ± 0.40, *p* < 0.0001). There were no significant difference between the groups of probable AD and the possible DLB (early: 2.36 ± 0.82, *p* = 0.0864; delayed: 2.25 ± 0.99, *p* = 0.0317) (Fig. 3). The group of patients with moderate/severe dementia consisted of 30 with probable AD, 18 with possible and 30 with probable DLB. In the moderate/severe dementia group, there were also significant differences in the H/M ratio between the groups of probable DLB (early: 2.06 ± 0.69, delayed: 1.90 ± 0.83) and probable AD (early: 2.65 ± 0.61, *p* < 0.01; delayed: 2.66 ± 0.80, *p* < 0.01). There were no significant difference between the groups of probable AD and possible DLB (early: 2.30 ± 0.68, *p* = 0.4686; delayed: 2.34 ± 0.86, *p* = 0.4076) (Fig. 3).

**Fig 4:**
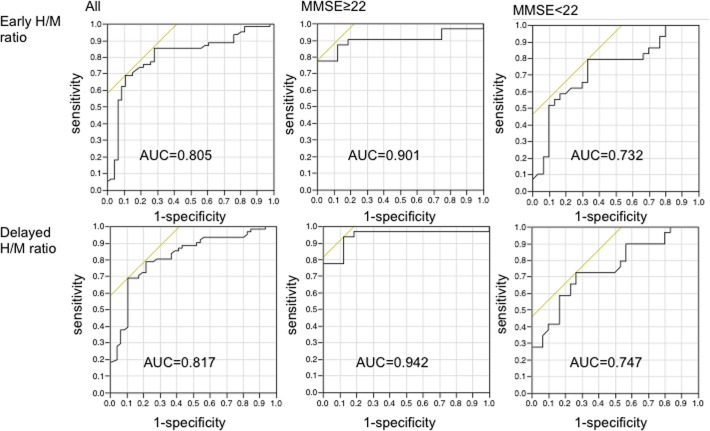
ROC curves for the detection of probable DLB from probable AD based on H/M ratio of each group. The area under the ROC curve of the early H/M ratio was 0.805 (*p* < 0.001) for the all patients group, 0.901 (*p* < 0.0001) for the mild dementia group, and 0.732 (*p* = 0.001) for the moderate/severe dementia group, whereas that for the delayed H/M ratio was 0.817 (*p* < 0.001), 0.942 (*p* < 0.0001), and 0.747 (*p* = 0.007), respectively. ROC: receiver operating characteristic. AUC: area under the curve.

When a ROC analysis was performed for discriminating probable DLB from probable AD groups, the area under the curve (AUC) of the early H/M ratio was 0.805 (*p* < 0.001) for the all patients group, 0.901 (*p* < 0.0001) for the mild dementia group, 0.732 (*p* = 0.001) for the moderate/severe dementia group, whereas that for the delayed H/M ratio was 0.817 (*p* < 0.001), 0.942 (*p* < 0.0001) and 0.747 (*p* = 0.0007), respectively (Fig. 4).

The sensitivity and specificity using cutoff values of the highest diagnostic accuracy based on ROC analysis are shown in Table 3. The all patients group had a sensitivity of 68.9% and a specificity of 89.1% at a cutoff value of 2.10 in both early and delayed H/M ratios. When applying the cutoff value of 2.10 to the delayed H/M ratio, the sensitivity and specificity for discriminating probable DLB from probable AD were 77.4% and 93.8%, respectively, in the mild dementia group. The moderate/severe dementia group, on the other hand, had a sensitivity of 59.6% and a specificity of 83.3% at a cutoff value of 2.10. No adverse events were noted during this study.

**Table 3:** Sensitivity and specificity of H/M ratio in differentiating between probable DLB and probable AD.

	Sensitivity (95% CI)	Specificity (95% CI)	Accuracy (95% CI)	PPV (95% CI)	NPV (95% CI)
Early H/M ratio (cutoff: 2.10)	68.9 (57.2–80.5)	89.1 (80.1–98.1)	77.6 (69.7–85.5)	89.4 (80.5–98.2)	68.3 (56.6–80.1)
Delayed H/M ratio (cutoff: 2.10)	68.9 (57.2–80.5)	89.1 (80.1–98.1)	77.6 (69.7–85.5)	89.4 (80.5–98.2)	68.3 (56.6–80.1)

PPV: positive predictive value; NPV: negative predictive value

## Discussion

### Diagnostic accuracy

This first multicenter study indicated that ^123^I-MIBG cardiac scintigraphy is a useful method to discriminate DLB from AD. The overall diagnostic accuracy for differentiating probable DLB from AD was 68.9% sensitivity and 89.1% specificity, and was particularly high in the mild dementia group showing a sensitivity of 77.4% and a specificity of 93.8%. This finding confirms and further extends findings of earlier single-site studies [13, 25]. The multicenter study using ^123^I-FP-CIT SPECT showed that mean sensitivity of 77.7% for detecting clinical probable DLB, with specificity of 90.4% for excluding non-DLB dementia, which was predominantly due to AD [26]. On the other hand, the recent clinicopathologic analyses showed that the DLB diagnostic criteria [15] had sensitivity of 85% and specificity of 73% for excluding non-DLB dementia [27]. Therefore, the sensitivity and specificity of ^123^I-MIBG cardiac scintigraphy are comparable to those of ^123^I-FP-CIT SPECT multicenter study, especially in mild dementia cases. The potential benefit in the diagnostic precision provided by ^123^I-MIBG cardiac scintigraphy is therefore predominantly in the specificity of case detection, which could be increased from a mean of 73% to 89.1% reported here.

### Variability in dementia severity

The reason of the variability of the sensitivity and specificity between mild and moderate to severe dementia groups is unclear. It was reported that extrapyramidal signs and hallucination occur frequently and progress in AD [28]. These confounding factors may affect the accuracy of diagnosis in moderate to severe dementia cases. The subclinical comorbid pathologies in patients with dementia also have influenced the results of the study [29]. This possibility needs to be evaluated through a long-term follow-up. Several studies reported that patients with DLB showed significantly lower ^123^I-FP-CIT uptakes in all striatal areas compared with AD patients with parkinsonism [30, 31]. These studies relevant to variability of symptoms indicate that adding ^123^I-FP-CIT SPECT to the ongoing protocol of the prospective study may also clarify this issue.

### Qualitative and quantitative assessment

^123^I-MIBG H/M ratio and visual interpretation showed comparative diagnostic accuracy for discriminating DLB and AD patients. To apply MIBG imaging to DLB patients in a number of hospitals, a quantitative approach is helpful to reduce inter-institutional variations even without interpretations of nuclear medicine specialists. The diagnostic accuracy based on early and delayed H/M ratios was also comparable. Our ROC analysis showed that the borderline of H/M ratio = 2.10 for both the early and the delayed H/M ratios can be practically used as optimal thresholds. Our method using semi-automatic regional setting and inter-institutional calibration contributed to obtain stable H/M ratios.

### Limitations

A limitation of our study design is that the gold standard for image validation was a clinical and not a neuropathological diagnosis. However, the clinical consensus panel technique has been shown to be accurate in a prospective diagnostic study with neuropathological confirmation, using the similar three reader system [9]. The consensus panel approach that we used is therefore justifiable.

A second potential limitation is that evaluation of cognitive fluctuation and rapid eye movement sleep behavior disorder were made by clinician’s impression and history of patients’ illness at each institution. In addition, dopamine transporter imaging was not available in Japan at that time. Therefore, low dopamine transporter binding in the basal ganglia as shown by SPECT or positron emission tomography imaging, which is a suggestive feature of DLB, was not used for the diagnosis of DLB in this study. These conditions may have caused the relatively low sensitivity of this study compared with previous studies [13, 25].

### Conclusions

This first multicenter study indicated that ^123^I-MIBG imaging can make a significant contribution to increasing the diagnostic accuracy of DLB. The technique is acceptable to patients, and the image reconstruction and the visual and automated ROI analysis are practical and sufficiently robust for use in multiple clinical settings. ^123^I-MIBG cardiac imaging seems to offer a significant advance in improving our ability to distinguish DLB from AD, especially in mild dementia cases.

## Supporting Information

S1 Table The list of approval by institutional review boards of all participating centers. (XLSX)
